# Characterization and regulation of an additional actin-filament-binding site in large isoforms of the stereocilia actin-bundling protein espin

**DOI:** 10.1242/jcs.143255

**Published:** 2014-03-15

**Authors:** Lili Zheng, Dina M. Beeler, James R. Bartles

**Affiliations:** 1 Department of Cell and Molecular Biology, Northwestern University Feinberg School of Medicine, Chicago, IL 60611 USA; 2 Hugh Knowles Center for Clinical and Basic Science in Hearing and Its Disorders, Northwestern University, Evanston, IL 60208 USA

**Keywords:** Espin, Actin, Bundle, Hair cell, Stereocilia, Ankyrin repeat, Myosin III, Autoinhibition

## Abstract

The espin actin-bundling proteins, which are produced as isoforms of different sizes from a single gene, are required for the growth of hair cell stereocilia. We have characterized an additional actin-filament-binding site present in the extended amino-termini of large espin isoforms. Constitutively active in espin 2, the site increased the size of actin bundles formed *in vitro* and inhibited actin fluorescence recovery in microvilli. In espin 1, which has an N-terminal ankyrin repeat domain, the site was autoinhibited by binding between the ankyrin repeat domain and a peptide near the actin-binding site. Deletion of this peptide from espin 1 activated its actin-binding site. The peptide resembled tail homology domain I of myosin III, a ligand of the ankyrin repeat domain localized with espin 1 at the tip of stereocilia. A myosin III tail homology domain I peptide, but not scrambled control peptides, inhibited internal binding of the ankyrin repeat domain and released the espin 1 actin-binding site from autoinhibition. Thus, this regulation could result in local activation of the additional actin-binding site of espin 1 by myosin III in stereocilia.

## INTRODUCTION

Parallel-actin-bundle-containing structures contain multiple classes of actin-bundling protein (Tilney et al., 1998; Bartles, 2000). An impressive example is hair cell stereocilia. Staircase-like arrays of stereocilia are detectors for hearing and the vestibular sense (Vollrath et al., 2007; Schwander et al., 2010; Richardson et al., 2011). Stereocilia contain a parallel-actin-bundle cytoskeletal scaffold and multiple actin-bundling proteins of the plastin–fimbrin, fascin and espin classes (Tilney et al., 1989; Zheng et al., 2000; Daudet and Lebart, 2002; Shin et al., 2010; Chou et al., 2011; Perrin et al., 2013; Shin et al., 2013). The espin actin-bundling proteins, which are the target of mutations that cause deafness and vestibular dysfunction (Zheng et al., 2000; Naz et al., 2004), are enriched in hair cell stereocilia (Zheng et al., 2000; Sekerková et al., 2006) and the microvilli of other sensory cells (Sekerková et al., 2004). Proteomic studies of stereocilia have detected espins to be present at lower levels than some other actin-bundling proteins (Shin et al., 2013). Nevertheless, the pronounced stereociliary defects observed in jerker mutant mice, which lack espin proteins because of a mutation in the espin gene (Zheng et al., 2000), suggest that espins are required to increase the diameter and length of the parallel actin bundle of stereocilia during morphogenesis (Sekerková et al., 2011).

Espins show no obvious resemblance to the other actin-bundling proteins of vertebrates. In addition, they differ from other actin-bundling proteins in that they are produced from a single gene in multiple isoforms that differ widely in molecular mass (∼28–91 kDa) owing to different sites of transcriptional initiation (Sekerková et al., 2004). Our earlier studies suggested that the ∼14 kDa C-terminal peptide shared by all espin isoforms, the actin-bundling module, is necessary and sufficient for actin bundling *in vitro* (Bartles et al., 1998). If this small fragment can bundle actin filaments, then why have different isoforms, and what roles do their distinct N-terminal segments play? Upstream of the actin-bundling module, there is a WH2 domain, which is believed to bind to monomeric actin, and different numbers of proline-rich domains, but the bulk of this sequence remains uncharacterized (Sekerková et al., 2004). The largest isoform, espin 1, also contains eight ankyrin-like repeats at its N-terminus (Bartles et al., 1996). This ankyrin repeat domain can bind to the tail homology domain I of myosin III (MYO3) (Salles et al., 2009). Through this interaction, espin 1 has been proposed to act as a cargo for MYO3A (Salles et al., 2009) and as a ‘crutch’ to support the movement of MYO3B (Merritt et al., 2012). Both motors are enriched in hair cell stereocilia and concentrated at their tips (Schneider et al., 2006; Salles et al., 2009; Walsh et al., 2011; Merritt et al., 2012; Shin et al., 2013).

Previously, we noted that a different region in the extended N-terminal segments of large espin isoforms appears to have actin-filament-binding activity (Chen et al., 1999). Specifically, a hexahistidine (His)-tagged fragment containing the central region (D339-V720) of the 837-residue rat espin 1 isoform bound filamentous actin (F-actin) with a *K*_d_ of ∼1 µM *in vitro*. When tagged with green fluorescent protein (GFP), this fragment also decorated F-actin-rich stress fibers in transfected fibroblastic cells. Through an examination of overlapping subfragments, this additional actin-filament-binding site, named the xAB (x for ‘extra’), was provisionally mapped to a 23-residue peptide, H459-K481 of rat espin 1, positioned far upstream of the other known actin-binding sites in espins (Chen et al., 1999). The xAB is present only in the larger espin isoform size classes, espin 1 and espin 2. Here, we characterize the xAB and show it has a major effect on the formation and dynamics of actin bundles and is subject to regulation involving autoinhibition with release by a peptide in MYO3.

## RESULTS

### The xAB binds actin filaments but not monomeric actin

**Fig. 1.**
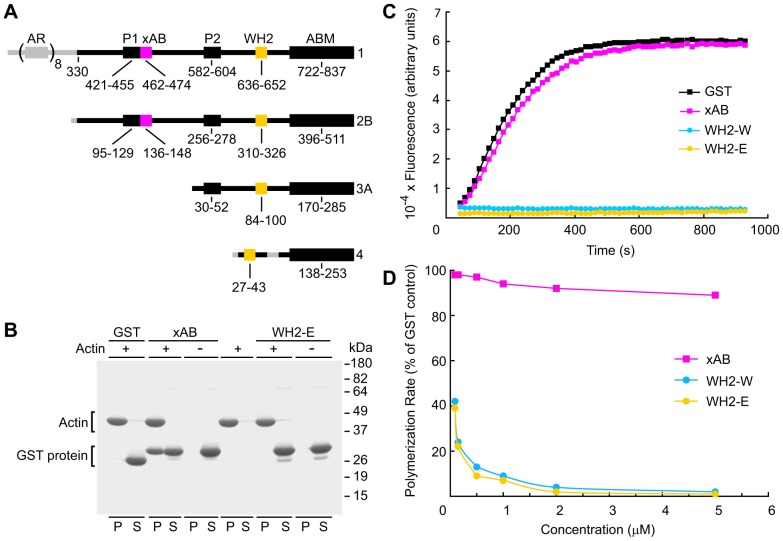
**Binding of the espin xAB to actin filaments.** (A) Stick-figure diagram showing representative examples of the different espin isoform size classes, highlighting their different N-terminal segments (left) and the position of the xAB in relation to other domains (ABM, actin-bundling module; AR, ankyrin-like repeat; P1 and P2, proline-rich domains 1 and 2). Segments in gray are unique to that isoform size class. Domain amino acid numbers are shown below. (B) Gel of co-sedimentation assays examining the binding of the designated GST fusion protein to actin filaments. P, pellet; S, supernatant; WH2-E, GST–espin WH2 domain. (C) Pyrene-actin polymerization assays examining the designated GST fusion protein (5 µM). WH2–W, GST–N–WASP WH2 domain; WH2–E, GST–espin WH2 domain. (D) Actin polymerization rate as a function of concentration of the GST fusion proteins shown in C. Means of two or three independent experiments are shown.

The position of the xAB in relation to other domains in representatives of the four different espin isoform size classes is depicted schematically in Fig. 1A. The number designates isoform size class, and the letter suffix specifies the splice variant (Sekerková et al., 2004). We focused initially on rat espin 2B, which lacks the ankyrin repeat domain (Fig. 1A). To characterize the xAB, we used a combination of *in vitro* and cell transfection assays. Binding of the xAB to F-actin was assayed by co-sedimentation (Chen et al., 1999). When a glutathione S-transferase (GST) fusion with peptide V130-K155 of rat espin 2B, which included the 23-residue provisional xAB region (Chen et al., 1999), was incubated with F-actin, a large fraction of the fusion protein (42±2%; mean ± s.d., *n* = 3) was recovered in the high-speed pellet with F-actin (Fig. 1B). By contrast, this protein remained in the supernatant in the absence of F-actin, and the GST control protein did not co-sediment with F-actin (Fig. 1B). Furthermore, a second control GST fusion with peptide S297-G329 of rat espin 2B, which included the espin WH2 domain (Fig. 1A), did not co-sediment with F-actin (Fig. 1B). Binding to monomeric actin was assayed by inhibition of pyrene-actin polymerization using GST fusion proteins (Zuchero et al., 2009). A GST fusion protein containing a known actin-monomer-binding peptide, E419-T456 of rat N-WASP, which included its second WH2 domain, showed a concentration-dependent inhibition of actin polymerization (Fig. 1C,D). The GST–espin WH2 domain construct had a similar effect (Fig. 1C,D). At the relatively high concentration of 5 µM, each GST–WH2 domain construct completely inhibited actin polymerization (Fig. 1C,D). By contrast, the GST–xAB construct had only a minor effect on actin polymerization, inhibiting the rate of polymerization by only 11±2% (mean ± s.d., *n* = 3) relative to the GST control at 5 µM (Fig. 1C,D). These results suggested that the xAB preferentially bound actin filaments.

### The xAB maps to a conserved 13-residue peptide

**Fig. 2.**
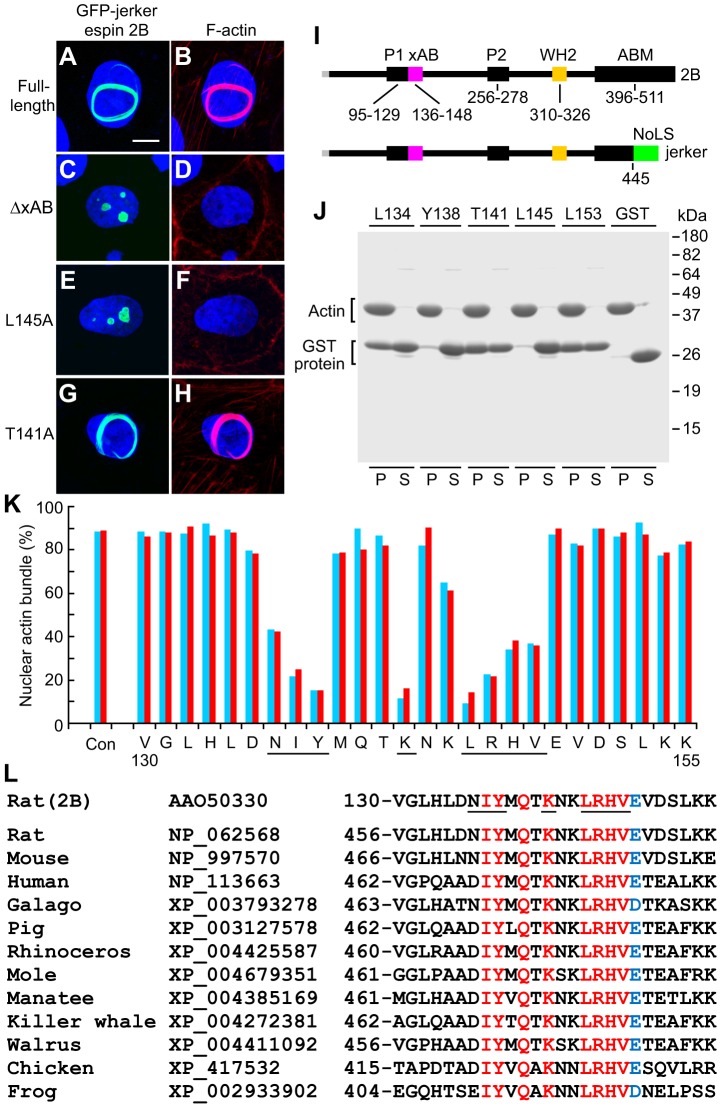
**Defining the xAB using nuclear actin bundle formation and actin filament-binding assays.** (A-H) Micrographs illustrating the most frequently obtained result in the nuclear actin bundle formation assay for the designated GFP–jerker espin 2B construct. F-actin is labeled with Texas Red-X-phalloidin (red), and nuclei are labeled with 4′,6-diamidino-2-phenylindole (blue). Scale bar in A: 10 µm. (I) Stick-figure diagrams of the espin 2B isoform showing the nucleolar localization sequence (NoLS, green) resulting from the jerker frameshift mutation and other domains. See Fig. 1A for domain abbreviations. (J) Gel of co-sedimentation actin filament-binding assays examining GST–xAB constructs (V130-K155, espin 2B) with the designated amino acid mutated to alanine. P, pellet; S, supernatant. (K) Graphical summary showing percentage of transfected cells with large nuclear actin bundles, instead of GFP-positive nuclear dots (nucleoli), upon expression of GFP–jerker espin 2B with the designated xAB amino acid mutated to alanine. Two independent experiments are shown, each examining >200 transfected cells. Underlined residues are required for activity. Con, GFP–jerker espin 2B control. (L) Amino acid sequences of the xAB region and GenBank Accession numbers for the rat espin 2B isoform and espin 1 isoforms of the designated species. Identical amino acids are indicated in red. Amino acids with only highly conservative substitutions are colored blue. Residues required for activity are underlined in the rat espin 2B sequence.

The boundaries and functionally important amino acids of the xAB were identified by scanning alanine mutagenesis. The results of quantitative assays examining nuclear actin bundle formation in transfected cells were confirmed by co-sedimentation of GST–rat xAB constructs with F-actin. As shown previously (Loomis et al., 2006), a large circular or C-shaped actin bundle formed in the nucleus when LLC-PK1 cells expressed cytomegalovirus promoter-driven GFP–espin-2B constructs bearing the jerker deafness mutation (Fig. 2A,B). This conspicuous actin bundle was detected in 90±2% of the transfected cells (mean ± s.d.; *n* = 7 experiments, each examining >200 transfected cells). The actin bundle formed in the nucleus because the jerker mutation brings about a frame shift that substitutes a nucleolar localization sequence for the second half of the espin actin-bundling module (Loomis et al., 2006) (Fig. 2I). Parts of the jerker espin 2B protein upstream of the nucleolar localization sequence were presumably involved in the growth and/or stabilization of the actin bundle. Consistent with a residual actin filament cross-linking function, GFP–jerker espin 2B colocalized with F-actin throughout the nuclear actin bundle (Fig. 2A,B). By contrast, GFP–jerker espin 2B with the V130-K155 xAB region deletion showed accumulation of the GFP protein in the nucleolus, but no nuclear actin bundle upon labeling with fluorescent phalloidin (Fig. 2C,D). This suggested that the xAB region was required for nuclear actin bundle formation.

The 26 amino acids in the V130-K155 xAB region of jerker espin 2B were individually mutated to alanine, and transfected cells expressing each GFP–jerker espin 2B construct were examined to determine whether they showed nuclear actin bundles or the negative outcome characterized by GFP-positive nucleoli. Some mutations – e.g. L145A – reduced nuclear actin bundle formation dramatically, so that bundles were seen in only 9–14% of the transfected cells (Fig. 2E,F,K). By contrast, constructs with other mutations – e.g. T141A – were similar to the control, producing nuclear actin bundles in 82–87% of the transfected cells (Fig. 2G,H,K). Using a decrease below 50% as the criterion to trigger assignment as a required amino acid, nuclear actin bundle-forming activity was found to span a 13-residue peptide: 136-NIYMQTKNKLRHV-148 (Fig. 2K). Three activity troughs pinpointed the amino acids required for activity: the first three residues (NIY), a central lysine (K142), and the last four residues (LRHV) (Fig. 2K, underlined). Accordingly, GST–xAB constructs (V130-K155) with these key residues mutated to alanine showed dramatically reduced *in vitro* F-actin-binding activity, relative to other alanine mutations and controls. Fig. 2J illustrates results for residues Y138 and L145. By contrast, alanine mutation of leucine residues positioned just outside of the 13-residue peptide (L134 and L153) or of the threonine residue in its middle (T141) did not decrease binding (Fig. 2J). In fact, the T141A and L153A mutations appeared to increase binding slightly (Fig. 2J). These results suggested that the nuclear actin bundle-forming assay reflected the F-actin-binding activity of the xAB. Seven of the eight residues required for activity were identical in espins from frog to human (Fig. 2L). The first residue, N136, showed only a partial reduction in nuclear actin bundle-forming activity upon mutation to alanine (Fig. 2K). This residue was conserved in rat, mouse and galago, but showed conservative substitution to aspartate in other mammals and chicken, and was substituted by glutamate in frog. The xAB showed no obvious sequence similarity to F-actin-binding peptides in the espin actin-bundling module or other actin-bundling proteins (Bartles et al., 1998; Volkmann et al., 2001; Jansen et al., 2011). Among the alanine mutations we examined, L145A showed a large decrease in activity (Fig. 2J,K), and constructs with this mutation were employed in other experiments.

### The xAB increases actin bundle size *in vitro*

**Fig. 3.**
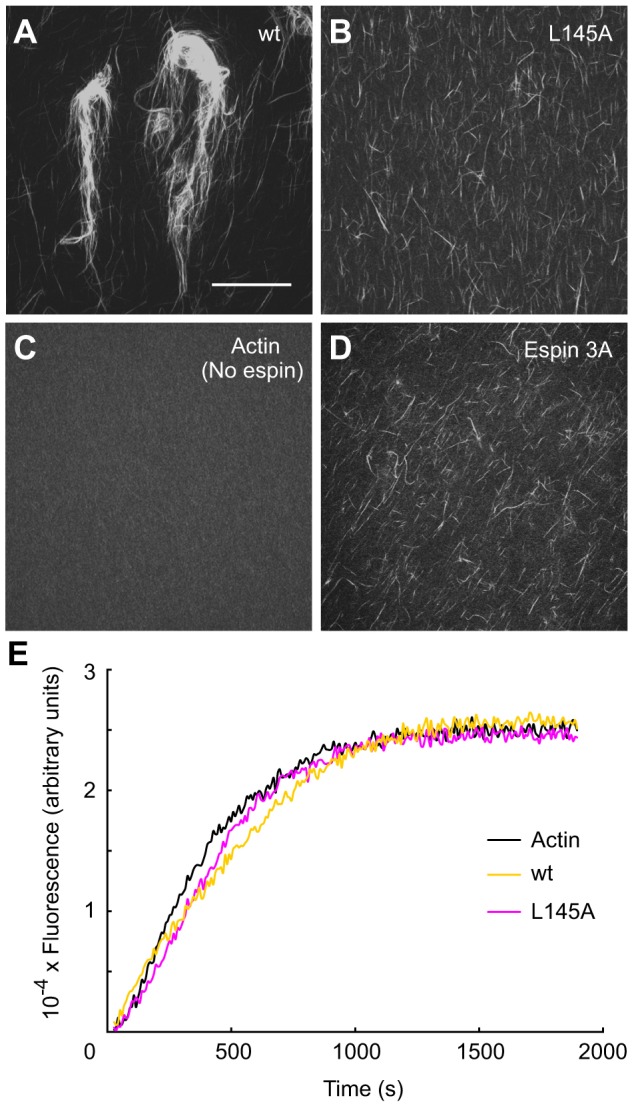
**Effect of the xAB on actin bundling and polymerization *in vitro*.** (A-D) Micrographs showing actin-bundling assays examining His-tagged wild-type (wt) espin 2B (A), His-tagged L145A-mutated espin 2B (B) or His-tagged espin 3A (D) at an espin∶actin molar ratio of 1∶20, compared with actin alone (C). Scale bar in A: 40 µm. (E) Spectrofluorimetric pyrene-actin polymerization assay time-courses examining His-tagged wild-type (wt) or His-tagged L145A-mutated espin 2B, at an espin∶actin molar ratio of 1∶20, compared with actin alone.

We detected a major role for the xAB in actin bundling by purified His-tagged espin proteins *in vitro*. Actin monomers containing 5% rhodamine-actin were mixed with purified His-tagged espin constructs at an espin∶actin molar ratio of 1∶20. Samples were incubated for one hour under conditions that support actin polymerization and bundling, and then examined by confocal microscopy. Fluorescence microscopy analysis allowed for a clearer discrimination of outcomes than low-speed co-sedimentation actin-bundling assays. Espin 2B formed large, polymorphic actin bundles (Fig. 3A). By contrast, espin 2B with the xAB-inactivating L145A mutation formed smaller, needle-like bundles of relatively uniform size (Fig. 3B). These smaller bundles were similar to those formed by espin 3A (Fig. 3D), which lacks the xAB (Fig. 1A). The large bundles formed by espin 2B appeared to comprise aggregates of smaller bundles, which could often be seen at frayed edges (Fig. 3A). Individual actin filaments containing rhodamine-actin were not well resolved under these conditions and contributed to diffuse background fluorescence (Fig. 3C). The difference in bundle size we observed with and without a functional xAB did not reflect a difference in actin polymerization; the L145A mutation had relatively little effect on the rate and extent of polymerization of pyrene-actin at this low espin-2B∶actin molar ratio (Fig. 3E). These results suggested that the xAB increased the valence and/or apparent affinity for F-actin, causing the formation of larger actin bundles.

### The xAB is required to inhibit actin fluorescence recovery in espin-containing microvilli

**Fig. 4.**
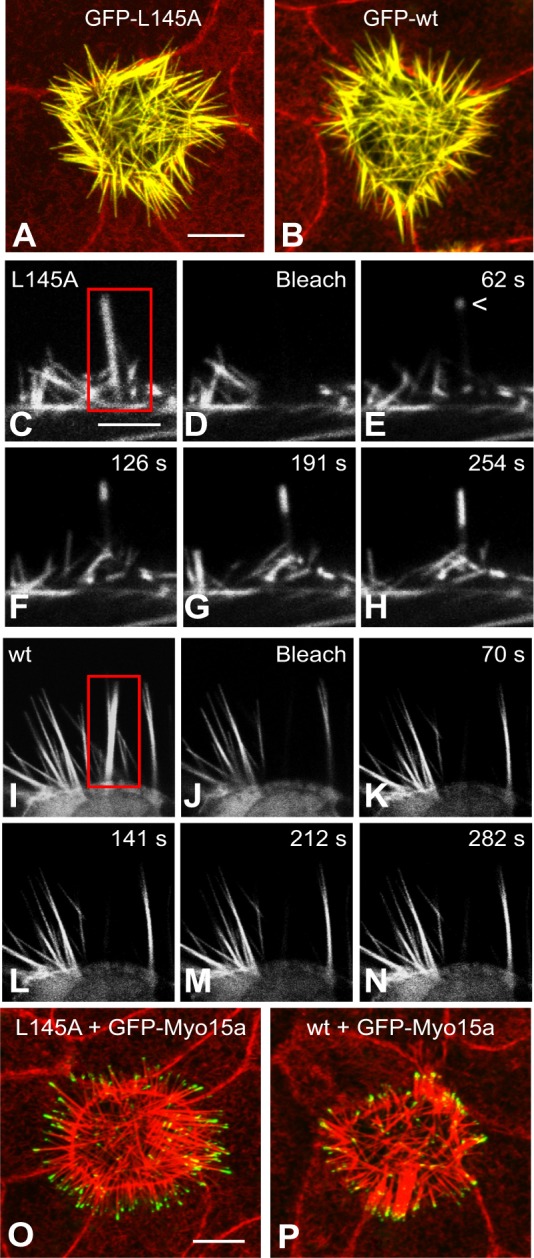
**Effect of the xAB on actin fluorescence recovery in espin-elongated microvilli.** (A,B) Micrographs showing the microvillar targeting and elongation in fixed LLC-PK1-CL4 cells expressing GFP–espin 2B with the L145A mutation (A) or wild-type (wt) GFP–espin 2B (B). F-actin labeling by Texas-Red-X-phalloidin is red. Scale bar in A: 10 µm. (C-N) Micrographs showing FRAP of GFP–actin in the long microvilli of living LLC-PK1-CL4 cells expressing untagged espin 2B with the L145A mutation (C-H) or untagged wild-type (wt) espin 2B (I–N). Red boxes in C and I indicate photobleached areas. Subsequent images were acquired immediately post-bleach (Bleach) or at recovery time shown in the upper right corner. Scale bar in C: 5 µm. (O,P) Micrographs showing localization of GFP–Myo15a (green) and F-actin (red) in the long microvilli of fixed LLC-PK1-CL4 cells expressing untagged espin 2B with the L145A mutation (O) or untagged wild-type (wt) espin 2B (P). Scale bar in O: 10 µm.

The xAB also had a major effect on actin dynamics in microvillar actin bundles. When expressed by transfection in LLC-PK1-CL4 epithelial cells, espins are efficiently targeted to microvillar actin bundles and increase the average bundle length from ∼1 µm to ∼7–9 µm (Loomis et al., 2003). This is illustrated for GFP-tagged espin 2B with and without the xAB-inactivating L145A mutation in Fig. 4A,B. The espins are concentrated throughout the long microvillar actin bundles (Fig. 4A,B) (Loomis et al., 2003; Sekerková et al., 2004; Zheng et al., 2010). Microvillar targeting and elongation require the espin actin-bundling module but not the xAB region (Loomis et al., 2003).

We took advantage of the increased spatial resolution provided by these long microvilli in our analysis of actin dynamics. Cells were co-transfected with untagged espin and GFP–β-actin plasmids, and 20–22 h later the long microvilli were examined by fluorescence recovery after photobleaching (FRAP). When long microvilli formed by espin 2B with the xAB-inactivating L145A mutation were examined by FRAP, there was a rapid recovery of GFP–actin fluorescence that began at the microvillar tip (Fig. 4C-H, arrowhead in E). This is the site of the ‘barbed’ or ‘+’ end of these microvillar actin filaments (Loomis et al., 2003). Over time, the fluorescent zone grew towards the microvillar base (Fig. 4E-H; supplementary material Movie 1). As microvilli did not elongate during recovery, this rapid, tip-to-base restoration of GFP–actin fluorescence was consistent with the turnover of existing actin filaments by ‘treadmilling’, in which unbleached (fluorescent) GFP–actin monomers added at the barbed end (tip) travel down through the filament, while bleached GFP-actin monomers dissociate from the pointed end (base). Evidence in support of actin treadmilling has been obtained for actin filaments (Fujiwara et al., 2002; Kuhn and Pollard, 2005) and cellular projections that contain a parallel actin bundle of relatively small diameter, known as filopodia (Mallavarapu and Mitchison, 1999), and microvilli (Tyska and Mooseker, 2002; Loomis et al., 2003). Although it is possible that additional actin filament nucleation also occurred, espin-elongated microvilli appear to be in a steady state 17–24 h after transfection (Loomis et al., 2003). We observed the rapid, tip-to-base recovery of GFP–actin fluorescence in 52 out of 53 of the espin 2B L145A specimens examined by FRAP (Table 1). It was also observed for long microvilli formed by the espin 2B V130-K155 xAB region deletion mutant and by espin 3A (Table 1), a shorter espin isoform that lacks the xAB (Fig. 1A).

**Table 1.**
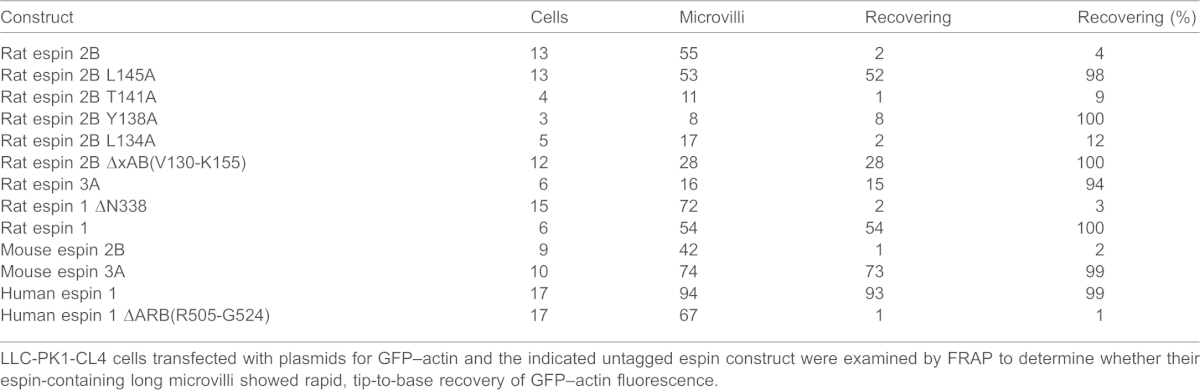
Espin-elongated microvilli showing rapid, tip-to-base recovery of GFP–actin fluorescence by FRAP

LLC-PK1-CL4 cells transfected with plasmids for GFP–actin and the indicated untagged espin construct were examined by FRAP to determine whether their espin-containing long microvilli showed rapid, tip-to-base recovery of GFP–actin fluorescence.

By contrast, the long microvilli formed by espin 2B with an intact xAB did not show a rapid recovery of GFP–actin fluorescence after photobleaching (Fig. 4I-N; supplementary material Movie 2). This lack of recovery was observed in 53 out of 55 of the espin 2B specimens examined (Table 1). Espin 2B has only a modest decelerating effect on actin polymerization in solution, suggesting that it does not cap the barbed end of filaments (Loomis et al., 2006). In fact, espin 3A, which is a more potent inhibitor of actin polymerization in solution than espin 2B (Loomis et al., 2006), supports actin fluorescence recovery in microvilli (Table 1). The absence of actin fluorescence recovery was also noted in long microvilli formed in response to espin 1 ΔN338, a truncated version of espin 1 missing the ankyrin repeat domain (M1-R338) (Table 1). The striking difference between espin 2B and espin 3A isoforms was also seen with mouse espins (Table 1). Four alanine mutations of the espin 2B xAB were compared. Mutation of the residues required for actin filament binding (Y138A, L145A) brought about prevalent tip-to-base GFP–actin fluorescence recovery, whereas other mutations (L134A, T141A) had only minor effects (Table 1). These results suggested that the F-actin-binding activity of the xAB was required to block actin fluorescence recovery in microvillar actin bundles and raised the possibility that the xAB was supplying additional, or stronger, F-actin cross-links that inhibited actin treadmilling flux. The actin bundles of microvilli that do not experience rapid actin fluorescence recovery were of the correct polarity, as shown previously by decoration with myosin subfragment 1 of microvilli elongated by espin 1 ΔN338 (Loomis et al., 2003). Confirmation came from our observation that a barbed-end-directed myosin motor, GFP–myosin XVa (Myo15a), accumulated at the tips of elongated microvilli whether formed by untagged espin 2B, with or without the L145A-mutated xAB (Fig. 4O,P).

### Localization of xAB-containing espin isoforms to the tip of stereocilia

**Fig. 5.**
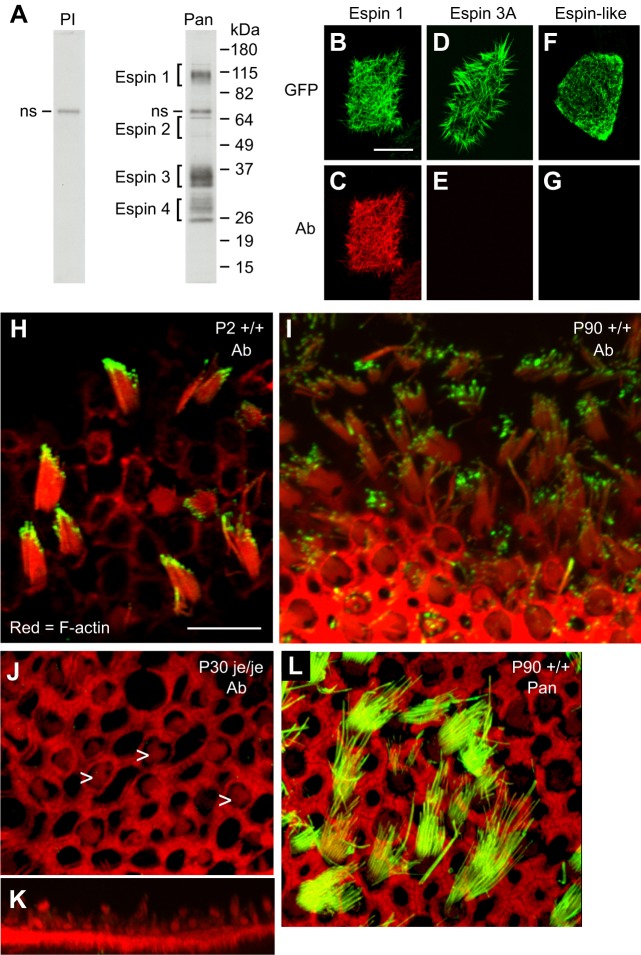
**Large espin isoforms in mouse utricle.** (A) Western blot of utricular maculae isolated from wild-type mice labeled with preimmune immunoglobulin (PI) or pan-espin antibody (Pan) showing the band clusters corresponding to the different espin isoform size classes. ns, non-specific band that was also detected on preimmune control. (B-G) Micrographs showing specificity of the antibody (Ab) against the large espin isoform when labeling LLC-PK1-CL4 cells expressing the designated GFP-tagged mouse protein. Scale bar in B: 10 µm. (H-K) Micrographs showing large espin isoform antibody (Ab) labeling of whole mounts of utricular maculae isolated from wild-type (*+/+*) or homozygous jerker (*je/je*) mice on the designated postnatal day (P). Antibody labeling is green, and F-actin labeling by Texas-Red-X-phalloidin is red. K is a volume snapshot of a portion of J showing a side-view of the collections of abnormally thin and short hair cell stereocilia in homozygous jerker mice (arrowheads in J). Scale bar in H: 10 µm. (L) Micrograph showing pan-espin antibody (Pan) labeling of whole mount of utricular macula isolated from a postnatal day 90 (P90) wild-type (*+/+*) mouse.

To assess the role of the xAB in stereocilia, we examined the large espin isoforms in the mouse utricle, a balance organ that senses movement and orientation. The utricle was selected because it is a concentrated source of hair cells, of which the stereocilia are of moderate length (Li et al., 2008), and the patch of hair cells (macula) is relatively easy to isolate by microdissection. Western blots of isolated mouse utricular maculae were labeled with affinity-purified pan-espin antibody. Band clusters corresponding to each espin isoform size class were present; however, the espin 1, espin 3 and espin 4 isoforms were detected at higher levels than the espin 2 isoforms (Fig. 5A). The complexity of the band clusters reflected the multiple splice variants that exist within each isoform size class (Sekerková et al., 2003; Sekerková et al., 2004; Sekerková et al., 2008), but protein modifications could also play a role. Blot scans showed approximately five times more espin 1 than espin 2 (Fig. 5A).

Recently, a polyclonal antibody to a peptide in the ankyrin repeat domain of espin 1 was reported to label the tips of hair cell stereocilia in mice (Salles et al., 2009). We had a concern that this antibody might be cross-reacting with the ankyrin-like repeats of other proteins, such as the espin-like protein, an espin paralog that is also present in stereocilia (Shin et al., 2013). The 1005-residue mouse espin-like protein (GenBank Accession number NP_001028464) shares a number of features with espins, including 10 ankyrin-like repeats at the N-terminus, a 10-residue proline-rich peptide and a 27-residue peptide that is 74% identical to the initial part of the espin actin-bundling module. Therefore, we re-examined this issue with an affinity-purified polyclonal antibody against the segment of rat espin 2B (M1-S216) that extends in the N-terminal direction beyond espin 3A, corresponding to peptide S330-S542 in rat espin 1 (Fig. 1A).

Antibody specificity in immunocytochemistry was tested by labeling transfected LLC-PK1-CL4 cells expressing GFP-tagged mouse proteins. The antibody reacted strongly with cells expressing mouse large espin isoforms (Fig. 5B,C), but not those expressing smaller mouse espin isoforms (Fig. 5D,E) or the mouse espin-like protein (Fig. 5F,G). The specificity of this antibody for large espin isoforms on western blots has been demonstrated previously (Sekerková et al., 2004). When this antibody was used to label whole mounts of mouse utricular maculae, the antibody staining (green) was concentrated at the tips of hair cell stereocilia at postnatal day 2 (Fig. 5H) and postnatal day 90 (Fig. 5I). The bright-red staining surrounding the hair cells in planes below the stereocilia corresponds to the massive circumferential F-actin belts that develop in neighboring supporting cells (Burns et al., 2008). The antibody did not label the abnormally thin and short stereocilia found in the utricle of homozygous jerker mice (Fig. 5J,K) (Sekerková et al., 2011), which do not accumulate espin proteins (Zheng et al., 2000). By contrast, the affinity-purified polyclonal pan-espin antibody, which recognizes all espin isoforms but not the espin-like protein, labeled the entire length of stereocilia (Fig. 5L). These results confirmed that, although espin isoforms are present throughout the stereocilium, the large espin isoforms, which consist mostly of espin 1, are concentrated at the tip. In addition, these results support the hypothesis that the barbed-end-directed MYO3 motors, which bind to espin 1, are responsible for its compartmentalization (Salles et al., 2009; Merritt et al., 2012).

### Autoinhibition of the xAB in espin 1

**Fig. 6.**
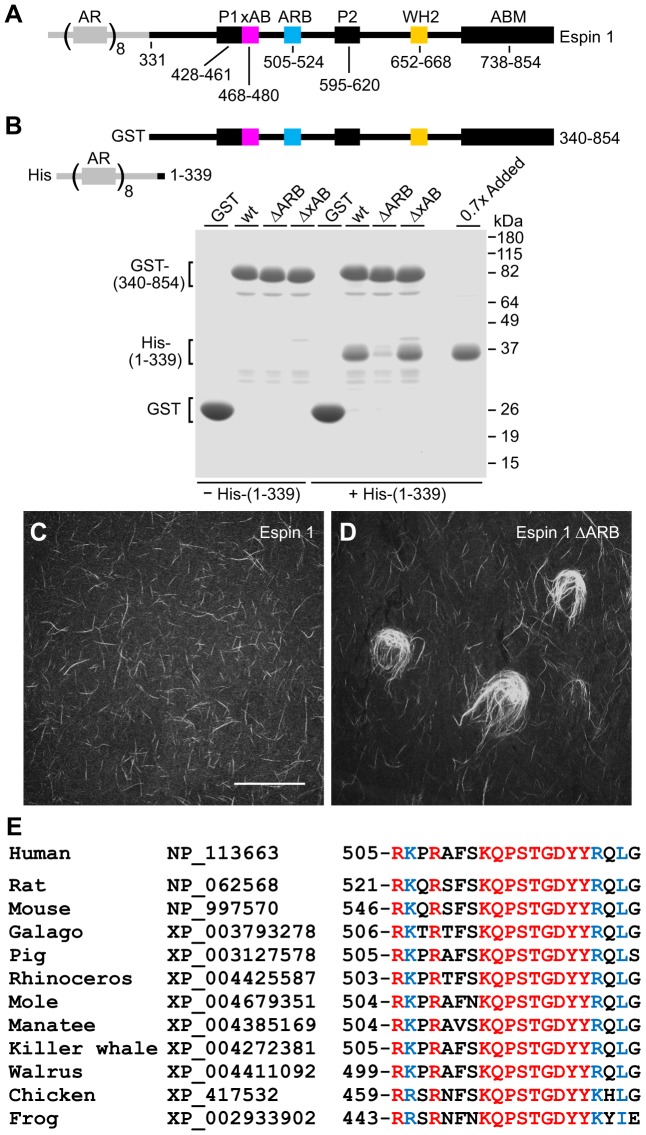
**Autoinhibition of the espin 1 xAB through an internal ankyrin repeat domain-binding site in large espin isoforms.** (A) Stick-figure diagram of human espin 1, showing the position of the 20-residue internal ankyrin repeat-binding site (ARB) in relation to other domains. (B) Stick-figure diagrams of the two tagged human espin 1 fragments used in the pull-down binding assays and gel of pull-down assays comparing the binding of the His–(1-339) human espin 1 ankyrin repeat domain construct to glutathione-beads loaded with GST or the designated GST–(340-854) human espin 1 fragment. The fragment was wild-type (wt) or missing the R505-G524 ARB peptide (ΔARB) or the V462-K487 xAB-containing peptide (ΔxAB). ‘0.7x Added’, sample of the His–(1-339) equal to 70% of that added to each binding reaction. (C,D) Micrographs showing actin-bundling assays examining His-tagged full-length human espin 1 (C) or His-tagged full-length human espin 1 missing the R505-G524 ARB peptide (D) at an espin∶actin molar ratio of 1∶20. Scale bar in C: 40 µm. (E) Amino acid sequences and GenBank Accession numbers of the ARB in espin 1 isoforms of the designated species. Identical amino acids are red. Amino acids with only very highly conservative substitutions are indicated in blue.

When we compared espin 1 with espin 2B, we found considerable differences. These experiments focused on human espin 1 (Fig. 6A) because it showed vastly improved yield and solubility as a His-tagged recombinant protein compared with espin 1 from mouse or rat (Chen et al., 1999). Unlike espin 2B, human espin 1 did not make large actin bundles *in vitro* (Fig. 6C), even though it included the xAB sequence (Fig. 2L). Instead, the human espin 1 formed small, needle-like bundles (Fig. 6C), like those formed by espin 2B with the xAB-inactivating L145A mutation (Fig. 3B). Similarly, human espin 1 differed from espin 2B in that it did not inhibit actin fluorescence recovery in microvillar actin bundles. When examined by FRAP, rapid, tip-to-base GFP–actin fluorescence recovery was observed in 93 out of 94 human espin 1 specimens examined and in all 54 rat espin 1 specimens (Table 1). These results suggested that the xAB of espin 1 was somehow masked.

As the main difference between espin 1 and espin 2B was the ankyrin repeat domain (Fig. 1A), we hypothesized that the ankyrin repeat domain blocked xAB activity by binding internally to a peptide near the xAB, in effect causing autoinhibition of the xAB. Consistent with this hypothesis, a His-tagged ankyrin repeat domain protein (M1-R339 of human espin 1) did bind to beads loaded with GST–human espin 1 D340-Y854 peptide, which included the fragment of espin 1 downstream of the ankyrin repeat domain (Fig. 6B). The GST–human 340–854 fragment bound 55±2% (mean ± s.d., *n* = 3) of the His–ankyrin repeat domain protein added to the reaction, but control beads loaded with GST did not bind to the protein (Fig. 6B). Through an examination of multiple truncation and deletion mutations of the GST–human espin 340–854 fragment, we identified a short peptide, R505-G524, that was required for binding the ankyrin repeat domain. This ankyrin repeat-binding peptide (ARB) was positioned only 24 amino acids downstream of the xAB (Fig. 6A). Deletion of the ARB from the GST–human espin 340–854 fragment decreased the percentage of the His–ankyrin repeat domain bound nearly eightfold to 7±2% (mean ± s.d., *n* = 3) (Fig. 6B). By contrast, deletion of the neighboring V462-K487 xAB-containing peptide had no discernible effect (Fig. 6B). As hypothesized, deletion of the ARB from the full-length human espin 1 (i.e. with the ankyrin repeat domain) released the xAB from autoinhibition. Deletion of the ARB changed human espin 1 such that it now formed large actin bundles *in vitro* (Fig. 6D) and inhibited actin fluorescence recovery in microvillar actin bundles (Table 1). These results confirmed that binding between the ankyrin repeat domain and ARB of espin 1 caused autoinhibition of the xAB *in vitro* and in cells.

### Activation of the espin 1 xAB by MYO3 peptide

**Fig. 7.**
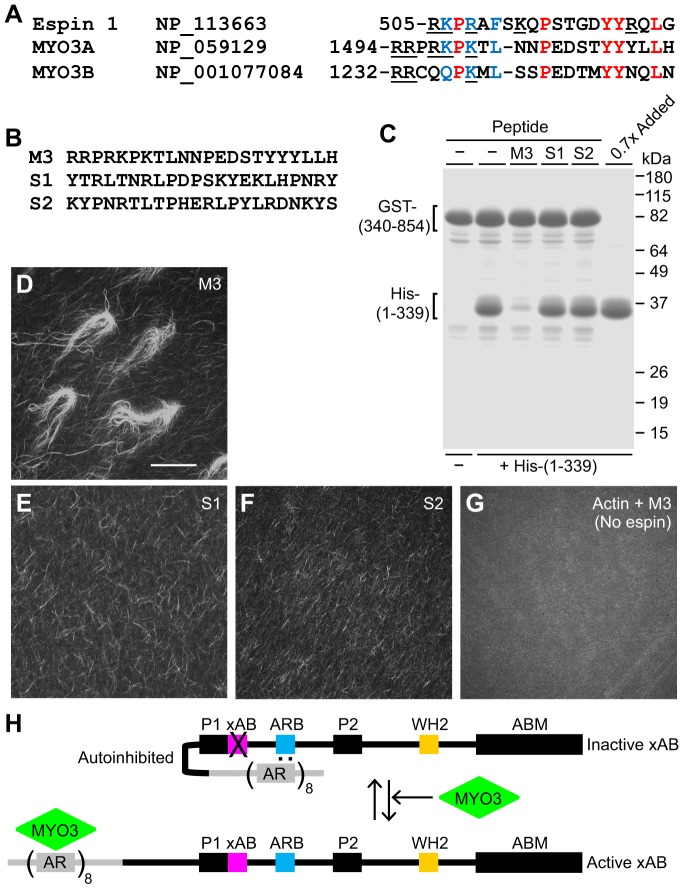
**Regulation of the espin 1 xAB by MYO3 peptide.** (A) An alignment of the internal ankyrin repeat-binding site (ARB) of human espin 1, the initial part of human MYO3A tail homology domain I that is sufficient to bind the human espin 1 ankyrin repeat domain and the related sequence in human MYO3B, highlighting identical residues (red) and very highly conservative substitutions (blue). Amino acids with positively charged side-chains are underlined. GenBank Accession numbers are included. (B) Synthetic peptides used in pull-down binding assays and actin-bundling assays, corresponding to the MYO3A tail homology domain I segment in A (M3) and two scrambled control peptides (S1 and S2). (C) Gel of pull-down assays examining the effect of the designated synthetic peptide at a concentration of 30 µM on the binding of the His–(1-339) human espin 1 ankyrin repeat domain construct to glutathione-beads loaded with the GST–(340-854) human espin 1 fragment (see Fig. 6B). ‘0.7x Added’, sample of the His–(1-339) construct equal to 70% of that added to each binding reaction. (D-G) Micrographs showing actin-bundling assays examining His-tagged full-length human espin 1 in the presence of 30 µM of the designated peptide (D-F) or examining 30 µM of M3 peptide in the absence of espin 1 (G). Scale bar in D: 40 µm. (H) Stick-figure showing the model proposed for the autoinhibition of the xAB in espin 1 by intramolecular interaction between the ankyrin repeat domain and the ARB (dots) and the activation of the xAB resulting from exposure to MYO3. Displacement of the ARB from the ankyrin repeat domain by the tail homology I domain of MYO3 releases the xAB from autoinhibition. See Fig. 1A for domain abbreviations.

The ARB is conserved among espins (Fig. 6E). The N-terminus of the ARB is enriched in positively charged amino acids and includes consecutive tyrosines towards the C-terminus. This resembled the initial 22-residues of the MYO3A tail homology domain I (Fig. 7A), which is sufficient to bind the espin 1 ankyrin repeat domain (Salles et al., 2009). The alignment in Fig. 7A also revealed two proline residues and two hydrophobic residues common between the proteins. MYO3B, which also binds the espin 1 ankyrin repeat domain (Merritt et al., 2012), contains a related peptide (Fig. 7A). At a concentration of 30 µM, the MYO3A peptide (M3) (Fig. 7B) reduced the binding of the His–ankyrin repeat domain to the GST–human espin 340–854 fragment from 55±5% to 8±3% (mean ± s.d., *n* = 3) (Fig. 7C). However, two scrambled control peptides (S1 and S2 in Fig. 7B) had no effect (Fig. 7C). This suggested that the MYO3 peptide could compete specifically with the ARB for binding to the espin ankyrin repeat domain.

Therefore, we examined whether the MYO3 peptide could relieve autoinhibition of the xAB in intact espin 1. When included with full-length human espin 1 in the actin-bundling assay, the MYO3A peptide (30 µM) caused human espin 1 to form large actin bundles (Fig. 7D), similar to those formed by the human espin 1 ARB deletion mutant (Fig. 6D) or by espin 2B (Fig. 3A). By contrast, when the scrambled control peptides were included, human espin 1 formed only small, needle-like actin bundles (Fig. 7E,F), similar to those formed by human espin 1 without added peptide (Fig. 6C). The MYO3A peptide did not cause the bundling of actin filaments in the absence of human espin 1 (Fig. 7G). This suggested that tail homology domain I of MYO3, an espin 1-binding protein that is also concentrated at the tip of stereocilia (Schneider et al., 2006; Salles et al., 2009; Walsh et al., 2011; Merritt et al., 2012), could reverse the autoinhibition of the xAB of espin 1 and allow the domain to function (Fig. 7H).

## DISCUSSION

Actin filament binding by the xAB had a considerable effect on espin activities. The espin actin-bundling module is already thought to contain two actin filament-binding sites (Bartles et al., 1998). Thus, the xAB could render espin 2 isoforms and activated espin 1 trivalent for actin filaments. This could help explain why espin 1 ΔN338, which contains the xAB but no ankyrin repeat domain, has an affinity for F-actin that is higher than that of the smallest espin isoform (*K*_d_, 70 nM versus 220 nM) (Chen et al., 1999). Alternatively, large espin isoforms could substitute the xAB for an F-actin-binding site in the actin-bundling module. As it shows no sequence similarity to the actin-bundling module or other actin-bundling proteins, the xAB might contribute a different type of actin-binding site that interacts differently with actin filaments.

The xAB appears to allow large espin isoforms to overcome an intrinsic limit on actin bundle diameter. The actin-bundling protein fascin is limited to forming actin bundles of only ∼20 filaments *in vitro*, possibly because of the energy needed to twist actin filaments in bundles (Claessens et al., 2008). Presumably, this explains the small, needle-like actin bundles we observed in response to espin 1 or espin 2B with the L145A mutation. The limit in fascin-actin bundle diameter can be overcome by including a second actin-bundling protein (Claessens et al., 2008). Adding an active xAB to espins also caused the formation of large actin bundles. This activity could be physiologically relevant because espins are required for stereocilia to undergo an approximately twofold increase in diameter during morphogenesis, and this widening likely involves assembling additional layers of actin filaments at the periphery of a pre-existing bundle (Sekerková et al., 2011). When slowed in jerker heterozygotes, stereocilium widening appears to proceed from base to tip (Sekerková et al., 2011). Perhaps espin 1, transported by MYO3, tracks the barbed end of actin filaments in these outer layers of the bundle during their elongation.

The xAB requirement for espins to inhibit actin fluorescence recovery in microvilli was remarkably robust. One possibility is that espins with an active xAB help produce a more stably crosslinked actin bundle that no longer allows for the ready flux of actin monomers through filaments during turnover. Hair cell stereocilia do not show rapid actin treadmilling (Zhang et al., 2012), and electron tomography reveals that chick utricular stereocilia contain an extensively crosslinked actin bundle consisting of ∼200 filaments (Shin et al., 2013). Presumably, the effects of the xAB would be manifested at the tip of stereocilia, where we found large espin isoforms to be concentrated. Although barbed-end capping by espins alone seems unlikely (Loomis et al., 2006), espins contain a WH2 domain, which raises the possibility of interactions with actin monomer (Paunola et al., 2002; Chereau et al., 2005) and the filament barbed end (Co et al., 2007).

The xAB was constitutively active in the espin 2 isoforms, the predominant isoforms in cerebellar Purkinje cells (Sekerková et al., 2003). However, we found more espin 1 than espin 2 in isolated mouse utricular maculae. The smaller espin 3 and espin 4 isoforms were also detected and presumably account for the intense pan-espin antibody labeling observed elsewhere in the body of stereocilia. The xAB of espin 1 proved to be inactive *in vitro* and in transfected cells. We propose that, in intact espin 1, the ankyrin repeat domain folds back to bind the ARB and either obstructs or unfolds the xAB, causing autoinhibition (Fig. 7H). The sequence of the ARB suggested a way to activate the espin 1 xAB using a physiologically relevant ligand. The ability to relieve xAB autoinhibition using a MYO3A tail homology domain I peptide suggested that MYO3 could activate espin 1 xAB locally in hair cell stereocilia. Thus, this regulation could provide a way to ensure that the xAB of espin 1 is turned on only in the proper subcellular compartment.

## MATERIALS AND METHODS

### Recombinant protein expression and purification

Chemicals were from Sigma-Aldrich (St Louis, MO). Mutations were introduced by PCR or restriction enzyme digestion and checked by DNA sequence analysis. Recombinant proteins were produced in *Escherichia coli* BL21 Star (DE3) (Life Technologies, Grand Island, NY). Rat espin 2B was formerly known as rat Purkinje cell isoform 1 (GenBank Accession Number AAO50330). Phenylmethylsulfonyl fluoride (1 mM) and protease inhibitor cocktail (P8849, Sigma-Aldrich) were added during lysis. GST fusion proteins were expressed using the pGEX-4T-2 vector and purified using glutathione-Sepharose 4B (GE Healthcare, Piscataway, NJ) in phosphate-buffered saline, 1 mM dithiothreitol, 1 mM NaN_3_, pH 7.4, with elution by 10 mM glutathione. Espin constructs with an N-terminal His-tag were expressed using the ProEX HTa or HTb vector (Life Technologies) and purified using Ni-NTA agarose beads (Qiagen, Valencia, CA) under non-denaturing conditions. Briefly, bacterial extracts prepared in 50 mM Tris-HCl, 10% (v/v) glycerol, 10 mM 2-mercaptoethanol, pH 8.5, were clarified by centrifugation for 30 min at 150,000 ***g***. Bead washes contained 20 mM Tris-HCl, 0.1 M KCl, 20 mM imidazole, 12.5 mM 2-mercaptoethanol, 10% (v/v) glycerol, pH 8.5. For His-tagged espins, one intermediate wash step included 1.5 M NaCl, and another included 0.1% (v/v) Triton X-100. For His-tagged human ankyrin repeat domain, 1.0 M KCl was substituted for the 1.5 M NaCl wash, and the Triton X-100 wash was excluded. Elution buffer included 200 mM imidazole. Freshly isolated recombinant proteins were dialyzed overnight into assay buffer and clarified by centrifugation at 150,000 ***g*** for 60 min at 4°C before use.

### Actin filament binding

Purified GST fusion proteins were incubated with preformed filaments of rabbit skeletal muscle actin (AKL99; >99% pure, Cytoskeleton, Denver, CO) (0.4 mg/ml actin) for 60 min at 37°C in 10 mM HEPES, 0.1 M KCl, 1 mM dithiothreitol, 0.5 mM ATP, 1 mM MgCl_2_, 1 mM NaN_3_, pH 7.4. Pellet and supernatant fractions obtained from centrifugation at 150,000 ***g*** for 60 min at 4°C were analyzed in Coomassie-Blue-stained SDS gels.

### Actin polymerization

Actin polymerization was assayed at 22°C by fluorescence using 5 µM actin with 4% pyrene-actin (Cytoskeleton) (365 nm excitation, 407 nm emission). The inhibition of actin polymerization by GST fusion proteins was examined in the wells of FLUOTRAC 200 96-well, flat-bottom, medium-binding polystyrene microplates (Greiner Bio-One, Monroe, NC) using a Safire^2^ plate reader (Tecan, Männedorf, Switzerland) (Neidt et al., 2008). Monomeric actin (10 µM) in 5 mM Tris-HCl, 0.2 mM CaCl_2_, 0.2 mM ATP, 0.5 mM dithiothreitol, pH 8.0, was converted to the Mg^2+^ form by adding one-tenth volume of 2 mM EGTA, 0.5 mM MgCl_2_, 5 mM Tris-HCl, pH 7.4. After 2 min, an equal volume of purified GST construct was added in 10 mM imidazole-HCl, 0.1 M KCl, 1.6 mM EGTA, 0.5 mM dithiothreitol, pH 7.4. The slope of the curve from 50 to 200 s was used to compare polymerization rates. Reactions examining His-tagged espin constructs substituted HEPES for imidazole and were performed in a quartz microcuvette in a PC1 spectrofluorimeter (ISS, Champaign, IL).

### Actin bundling

Actin (1 mg) and 40 µg of rhodamine-rabbit skeletal muscle actin (Cytoskeleton) were hydrated together in 5 mM Tris-HCl, 0.2 mM CaCl_2_ 0.2 mM ATP, 0.5 mM dithiothreitol, pH 8.0, overnight and centrifuged at 150,000 ***g*** for 20 min at 4°C. In rapid succession, supernatant was diluted to an actin concentration of 5 µM in 10 mM HEPES, 0.1 M KCl, 1 mM dithiothreitol, 3 mM NaN_3_, pH 7.4, containing 2 mM MgCl_2_ and 1 mM ATP, and an equal volume of 0.25 µM purified His-tagged espin construct in the same buffer minus ATP and MgCl_2_ was added. Samples were mixed and incubated for 60 min at 37°C with gentle agitation at intervals of 15 min. Aliquots (∼6 µl) were delivered onto microscope slides using a pipet tip, trimmed to increase the opening diameter to ∼1.5-mm, and imaged using the ×100 objective on the LSM510 META laser scanning confocal microscope (Carl Zeiss, Thornwood, NY). Z-stacks are shown.

### Pull-down

His-tagged ankyrin repeat domain (M1-R339 of human espin 1) was incubated with glutathione-Sepharose beads preloaded with GST–human espin 340–854 fragment in 5 mM HEPES, 10 mM imidazole, 0.1 M KCl, 1 mM dithiothreitol, 3 mM NaN_3_, pH 7.4, for 60 min at 22°C. Beads were washed five times at 4°C by centrifugation at 14,000 ***g*** for 30 s, and pellet fractions were analyzed using Coomassie-Blue-stained SDS gels.

### Synthetic peptides

Some assays included high purity grade (>95%) custom synthetic peptides (GenScript, Piscataway, NJ). Peptides were dissolved in assay buffer and clarified by centrifugation at 150,000 ***g*** for 60 min before use.

### Cell transfection

LLC-PK1 cells (ATCC, Manassas, VA) and LLC-PK1-CL4 cells (Zheng et al., 2010) were cultured at 37°C in Dulbecco's Modified Eagle Medium (DMEM) alpha (Life Technologies) containing penicillin and streptomycin and 5% or 10% (v/v) fetal bovine serum (Life Technologies), respectively (Zheng et al., 2010). Cells in 35-mm dishes were transfected for 3.5 h with DMEM alpha containing 6 µl of Lipofectamine (Life Technologies) and 1.8 µg or 2.7 µg (co-transfection) of plasmid DNA and examined 20–22 h later. To assay for nuclear actin bundle formation (Loomis et al., 2006), LLC-PK1 cells plated on coverslips were transfected with jerker espin 2B constructs in the pEGFP-C2 vector (Life Technologies). The jerker espin 2B constructs were chimeras consisting of the first 445 residues of rat espin 2B, which included the xAB, and the last 66 residues of jerker mouse espins, which included the nucleolar localization signal introduced by the jerker mutation (Loomis et al., 2006). Cells were fixed with 2% (w/v) formaldehyde in phosphate-buffered saline, pH 7.4, and processed in the same buffer containing 0.01% (w/v) saponin. Cells were quenched with 0.25% (w/v) ammonium chloride, permeabilized for 30 s with ice-cold 0.1% (v/v) Triton X-100, labeled with Texas-Red-X phalloidin (Life Technologies) and 4′,6-diamidino-2-phenylindole (Life Technologies) and examined using a ×40 objective on a Zeiss Axioplan 2 fluorescence microscope, or imaged using a ×100 objective on the LSM510 META confocal microscope. Cells were scored for the presence of GFP- and Texas-Red-X-phalloidin-labeled nuclear actin bundles (positive) or GFP-labeled nucleolar dots with no Texas-Red-X-phalloidin-labeled actin bundle (negative) in two independent experiments, each examining >200 transfected cells on duplicate coverslips. To examine espin targeting and microvillar elongation, LLC-PK1-CL4 cells were transfected with pEGFP-C2 vector espin constructs, fixed with formaldehyde and labeled with Texas-Red-X-phalloidin (Loomis et al., 2003). To examine the polarity of microvillar actin bundles, LLC-PK1-CL4 cells were co-transfected with untagged espin plasmid construct in the pcDNA3 vector and GFP–Myo15a plasmid (Zheng et al., 2010), fixed with formaldehyde and labeled with Texas-Red-X-phalloidin.

### FRAP

LLC-PK1-CL4 cells were plated on No. 0 uncoated glass-bottom dishes (P35G-0-20-C; MatTek Corp., Ashland, MA) and co-transfected with equal amounts of untagged espin plasmid construct in the pcDNA3 vector (Life Technologies) and pEGFP–human beta-actin (Life Technologies). FRAP was performed 20–22 h after transfection in Leibovitz's L-15 medium (Life Technologies) containing 10% (v/v) fetal bovine serum at 37°C using a temperature-controlled stage. Initial experiments were conducted using a ×100 objective on the LSM510 META confocal microscope. Photobleaching was performed with the 488 nm laser at full power with a tube current of 6.5 A for 25–30 iterations. Post-bleach images were acquired at the designated intervals. Later experiments were carried out using a total internal reflection fluorescence ×100 NA1.49 objective (Nikon, Melville, NY) on a CSU-X1 spinning disk confocal (Andor, South Windsor, CT) equipped with FRAPPA module driven by Andor IQ2 software. The 488 nm laser was set at 100% for photobleaching at 50 µs pixel dwell and two iterations. Images were taken at 20 s intervals using an Andor iXon EMCCD camera and used to make QuickTime movies. For clarity, analysis was conducted on espin-elongated microvilli, ∼7–9 µm long, that extended out over the apical surface of a neighboring untransfected cell.

### Antibodies and immunolabeling

Antibodies were affinity purified or absorbed (depleted) using recombinant proteins covalently coupled to cyanogen-bromide-activated Sepharose 4B (Sigma). Pan-espin antibody was raised in rabbits against purified rat espin 2B protein, affinity purified on columns of rat espin 2B-Sepharose 4B (Sekerková et al., 2008) and absorbed with Sepharose 4B beads coupled with a GST fusion of peptide E354-D459 from the mouse espin-like protein. Antibody specific for large espin isoforms substituted Sepharose 4B coupled with His-tagged rat espin 2B P217-L511 peptide for the final absorption step. Antibody specificity was tested using LLC-PK1-CL4 cells transfected with pEGFP-C vector plasmid DNA encoding the designated mouse espin isoform or mouse espin-like protein and processed as above, except that sequential incubations with antibody and Texas-Red-X-labeled goat anti-rabbit F(ab′)_2_ (Jackson ImmunoResearch Labs, Bar Harbor, ME) were substituted for the incubation with Texas-Red-X-phalloidin. Utricular maculae were dissected from the inner ears of wild-type or homozygous jerker mice of the CBA/CaJ strain (Sekerková et al., 2011) and labeled as described above, except permeabilization was with 0.5% (v/v) Triton X-100 for 5 min, and 5% (v/v) goat serum was included in a 10 min wash before the overnight incubation with primary antibody at 4°C. Images were acquired using the ×60 objective on the Nikon A1R laser scanning confocal microscope. Image acquisition and 3D rendering were performed using Nikon Elements software. For western blotting, samples containing six utricular maculae were homogenized in 1 mM phenylmethylsulfonyl fluoride and protease inhibitor cocktail (P8849, Sigma-Aldrich), heated for 3 min at 100°C in SDS gel sample buffer, resolved by SDS gels, transferred electrophoretically to nitrocellulose in the presence of 20% (v/v) methanol and 0.4% (w/v) SDS and labeled with affinity-purified pan-espin antibody, followed by anti-rabbit ECL western blotting detection reagents (GE Healthcare).

## Supplementary Material

Supplementary Material
