# Supplementary material for: Characterization and regulation of an additional actin-filament-binding site in large isoforms of the stereocilia actin-bundling protein espin
Source: J Cell Sci. 2014 Mar 15;127(6):1306–17. doi: 10.1242/jcs.143255 (PMC3953818; doi:10.1242/jcs.143255)
Supplement: Supplementary Material [file supp_127_6_1306__index.html]

Characterization and regulation of an additional actin-filament-binding site in large isoforms of the stereocilia actin-bundling protein espin — Supplementary Material 

# Characterization and regulation of an additional actin-filament-binding site in large isoforms of the stereocilia actin-bundling protein espin

## JCS143255 Supplementary Material

**Files in this Data Supplement:**

- **Supplementary Material**
