# Supplementary material for: Characterization and regulation of an additional actin-filament-binding site in large isoforms of the stereocilia actin-bundling protein espin
Source: J Cell Sci. 2014 Mar 15;127(6):1306–17. doi: 10.1242/jcs.143255 (PMC3953818; doi:10.1242/jcs.143255)
Supplement: Supplementary Material [file supp_127.6.1306_JCS143255.pdf]

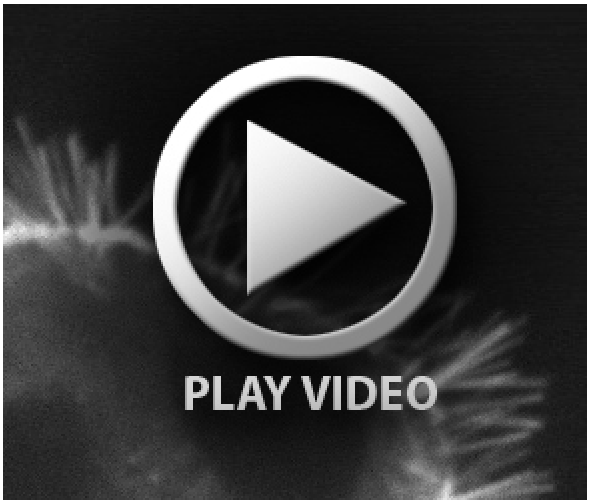

Movie 1. FRAP analysis of GFP-actin in long microvilli containing espin 2B L145A. Movie illustrating the rapid tip-to-base recovery of GFP-actin fluorescence observed when the espin xAB is inactive. The frame rate was increased by a factor of 70.

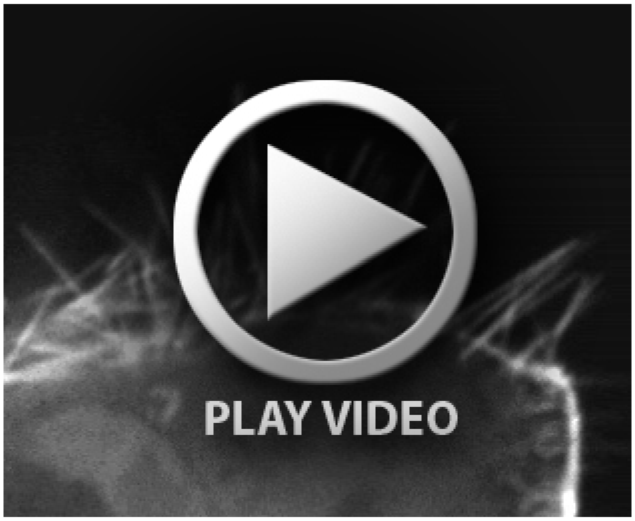

Movie 2. FRAP analysis of GFP-actin in long microvilli containing espin 2B. Movie illustrating the absence of rapid tip-to-base GFP-actin fluorescence recovery observed when the espin xAB is active. The frame rate was increased by a factor of 70.
